# Comparison of Projected and Actual Outcomes of the HIV/AIDS Epidemic in Malawi, 1990–2000

**DOI:** 10.1371/journal.pone.0006806

**Published:** 2009-08-27

**Authors:** Suja S. Rajan, David C. Sokal

**Affiliations:** 1 University of North Carolina, Chapel Hill, North Carolina, United States of America; 2 Scientist, Behavioral and Biomedical Research, Family Health International, Research Triangle Park, North Carolina, United States of America; University of Swansea, United Kingdom

## Abstract

**Background:**

In 1992 Liomba et al used HIV-AIDS prevalence data from 1980 to 1990 and projected the adult HIV prevalence and the impact of AIDS for the years 1991 to 2000, under high and low HIV incidence scenarios, using EpiModel, DemProj and the AIDS Impact Model. This report compares the actual outcomes of the HIV-AIDS epidemic in Malawi from 1991 to 2000 with projections made by Liomba et al.

**Methods and Findings:**

Due to the lack of data on rural HIV prevalence in 1992, the prevalence estimates for the years 1980 to 1990 used by Liomba et al were higher than the now published prevalence. We re-estimated the projections for 1991 to 2000 based on more recent estimates of 1980 to 1990 HIV-AIDS prevalence using the Spectrum modeling software, and compared the old and new projections with the actual epidemic figures reported by NAC. The original projections made by Liomba et al and the adjusted projections made in this report are both very similar to the observed course of the AIDS epidemic reported by National AIDS Commission. Unfortunately for Malawi the epidemic seems to have followed the high scenario of HIV incidence.

**Conclusion:**

The course of the epidemic in Malawi and the social and economic devastation caused by it, despite the presence of reasonably good projections and prevention recommendations by Liomba et al, highlights the need for better co-ordination between research and practice. It also emphasizes the need to examine the difficulties faced by countries like Malawi to act more rapidly and implement effective prevention measures in the face of an epidemic.

## Introduction

Malawi is one of the most AIDS-affected nations of the Sub-Saharan region, and ranks among the top 10 AIDS-afflicted countries of the world [1]. Estimates of the HIV-AIDS epidemic in Malawi published by National AIDS Commission (NAC) and other sources consistently demonstrate the gravity of the epidemic's effects [2]–[8]. Malawi has a current HIV prevalence of approximately 14.1% among adults, 25% among urban young adults (14–45 years of age), and 20% among antenatal clinic attendees [3]–[8]. Almost one million people of all ages live with HIV-AIDS in a national population of 12.3 million. With an estimated 110,000 new cases of HIV infection, 70,000 new cases of AIDS, and 80,000 deaths each year, AIDS is the leading cause of death among adults in Malawi [1].

In 1992, Liomba et al, including one of us (David Sokal) used an epidemiologic modeling approach to project the course of the AIDS epidemic in Malawi, including projections of adult HIV prevalence, annual adult AIDS deaths, number of AIDS orphans, number of tuberculosis patients, and total population size for the years 1991 to 2000 (Table 1). These projections were summarized in a monograph that was printed by Malawi's Ministry of Health, “Preventing AIDS in Malawi: A Policy Maker's Information Booklet.” It included recommendations for interventions (which included partner reduction, condom use, and improved STD control) to prevent and alleviate the AIDS epidemic [9]. Given the devastating HIV epidemic in Malawi it is useful to examine the accuracy of these projections, and to understand the importance of using epidemiologic models to guide policy making and to avert worse-case scenarios. In this brief communication, we re-visit those projections and compare them with the actual course of the epidemic in order to establish the validity of those projections. The comparison aims to emphasize the relevance of epidemiological projections, and the importance of taking them into account while designing and implementing preventive healthcare programs.

**Table 1:** Original Projections, Adjusted Projections and Actual NAC estimates for the year 2000.

	Original Projections for 2000 from the Liomba et al monograph (1992)		Adjusted Projections for 2000 after correcting for the prevalence from 1980 to 1990, based on the 2003 NAC estimates		Actual Estimates reported by NAC	
	High incidence scenario	Low incidence scenario	High incidence scenario	Low incidence scenario	Year 2000	Year 2003
**Adult (>15 years) HIV Prevalence (%)**	18.75%	10.10%	13.88%	5.23%	11.81%	12–17%*
**Annual AIDS Deaths**	65,000	42,000	53,430	41,440	70,000	80,000
**Number of AIDS orphans** **	365,000	275,000	314,940	283,510	342,000	384,000
**Number of Tuberculosis patients**	25,000	19,000	28,840	23,920	27,700	30,000
**Population size (in million)**	10.8	11.5	11.39	11.47	10.84	11.7

* These values are for adults aged 15–49 years.

** NAC estimated values for AIDS orphans were computed by multiplying total number of orphans estimated by NAC in 2000 by estimated percentage of orphans due to HIV/AIDS.

## Methods

### Epidemiological models used for the AIDS projections in the monograph

Liomba et al used EpiModel [10], Demproj, and the AIDS Impact Model (AIM) to produce the projections. DemProj is a demographics projection package with a long history of use that was modified at the beginning of the AIDS epidemic to incorporate estimates of HIV prevalence and HIV-AIDS mortality. AIM uses estimates of HIV prevalence and HIV-AIDS mortality to project the impacts of the AIDS epidemic. DemProj and AIM have now evolved and become subsumed into the Spectrum modeling package [11]. EpiModel is a simple spreadsheet that has been replaced by a more sophisticated approach, the Estimation and Projection Package (EPP) [12], which can export data for use in Spectrum. These three models fall under the category of type II HIV-AIDS models based on Chin's classification of AIDS projections models [10]. Spectrum is a suite of modules that now includes various other policy-oriented models in addition to AIM and Demproj. Both Spectrum and the Estimation and Projection Package are available on the UNAIDS web site, UNAIDS.org, and the Futures group website [11].

### Methodological assumptions used in the monograph

Liomba et al used EpiModel to estimate incidence levels in Malawi, based on the prevalence data available from 1980 to 1990. They estimated that the HIV incidence in Malawi was negligible between 1980 and 1984 but rapidly increased to an average of about 2% per year between 1985 and 1990. Based on this incidence estimation, they made various AIDS impact projections for high and low scenarios from 1991 to 2000 using Demproj and AIM (Table 1). In the high scenario the incidence rate was assumed to continue at 2% between 1991 and 2000, and in the low scenario the incidence rate was assumed to be 1% between 1991 and 2000 due to a reduction in the spread of infection compared to the early years of the epidemic. The high and low scenarios were designed to provide a range of likely health impacts for Malawi without an intervention. In the “Interventions: What can be done” section of the Liomba et al monograph, the potential benefits of preventive interventions (like partner reduction, condom use, and improved STD control) were illustrated using outputs from simulation modeling of a generic, urban, heterosexual epidemic [13].

### Adjustments made to the projections in the monograph to reflect the most current information

**Figure 1:**
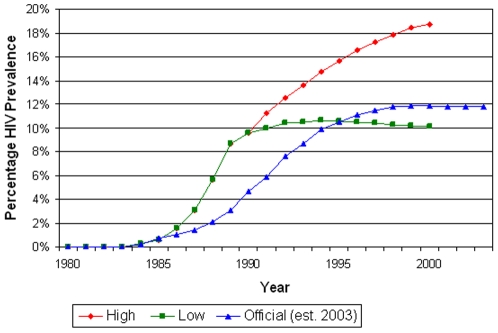
Original 1992 HIV prevalence projections for the high and low scenarios and 2003 NAC official estimates.

When we compared the prevalence estimates from the monograph to those reported by the NAC [2], we realized that the baseline prevalence used by the monograph for the years 1980 to 1990, on which the projections for 1991 to 2000 were based, were consistently higher than the now accepted estimates reported by the NAC (Figure 1). This difference is probably because in 1992 most HIV prevalence data were based on urban samples, and Liomba et al had to make assumptions about rural prevalence based on very sparse data. An over-estimate of rural HIV prevalence is probably the main reason for the monograph's overestimates of Malawian national prevalence for the years 1980 to 1990.

To better evaluate the accuracy of the projection methods we re-estimated the predictions in the monograph using the NAC reported prevalence from 1980 to 1990. We first linearly adjusted the predicted prevalence in the monograph, to account for the lower actual prevalence from 1980 to 1990. After this we re-computed the projections listed in table 1 by applying the incidence rates of 2% and 1% for the high and low scenarios respectively between 1990 and 2000, and we used Spectrum to project AIDS cases and other impacts. The tuberculosis incidence was also re-adjusted to match the current NAC estimates before performing the re-computations.

Since the NAC reported HIV prevalence for people between the years 15–49, and the monograph projected prevalence for adults 15 years and above, we adjusted the NAC values for all adults above the age 15 by using population weights, in order to make it comparable with the monograph's projected prevalence. Our re-estimated projections are hereafter referred to as “adjusted projections” and the monograph's projections are referred to as “original projections.” A copy of the original monograph is available upon request.

## Results

**Figure 2:**
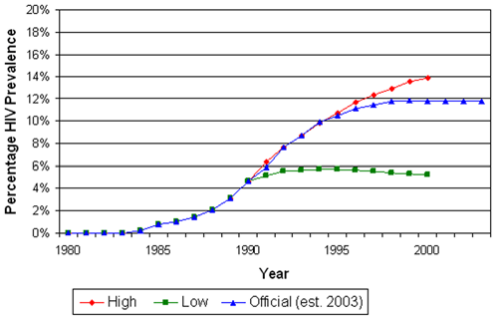
Adjusted 1992 HIV prevalence projections for the high and low scenarios and 2003 NAC official estimates.

The original projections, the adjusted projections and the NAC reported figures for the year 2000 are presented in Table 1. Figure 2 depicts the NAC's reported HIV prevalence [2] along with the adjusted projections. The reported prevalence lies very close to the high scenario after 1990 but with somewhat of a plateau after 1995.

The original HIV prevalence projection was 18.75% under the high scenario, and 10.1% under the low scenario (Table 1), for all adults over 15 years of age in the year 2000. The adjusted projections for the year 2000 were 13.88% and 5.23% under the high and low scenarios respectively. The adult HIV prevalence according to the NAC [2] was nearly 14% in 2000, for adults 15–49 years of age, and thus about 11.81% for adults above the age of 15 years. It was estimated to be between 12 to 17% for adults between the years of 15–49, as of 2003.

Regarding impacts of the epidemic in the year 2000 on: (a) annual AIDS deaths, (b) numbers of AIDS orphans, (c) number of TB patients, both the original and adjusted high incidence projections for the year 2000 are very close to recent estimates by the NAC [2]. The conclusion that population size would continue to grow was correct, and the population size projections for 2000 are close to the NAC estimates and slightly lower than the US Census Bureau estimate of 11.6 million [14].

## Discussion

Behavioral and biologic surveillance data and HIV-AIDS projections should help guide policymakers and public health officials to identify geographic areas or populations with higher incidence or prevalence; and help target, design and implement effective interventions. While model-based projections of AIDS cases were sometimes criticized as being alarmist or as exaggerating the dangers from HIV, the 1992 monograph on Malawi was accurate in its projections, despite the limited prevalence data available (Table 1). Although the original projections are slightly higher than the adjusted projections, due to the less accurate baseline prevalence used by the monograph for the years 1980 to 1990, the two projections are not very different and are very close to the recent estimates of the NAC [2]. The adjusted results illustrate that in the presence of reliable prevalence estimates, projections of the epidemic using relatively simple epidemiological models can provide good estimates of the future state of the epidemic even at a relatively early stage of a generalized epidemic. Unfortunately for Malawi, the comparison of both the original projections by Liomba et al and the adjusted projections with the actual outcomes demonstrates that the epidemic proceeded along the predicted high scenario of HIV incidence (Table 1).

As mentioned above, the Liomba et al monograph also included results from a generic simulation model, illustrating a reduction in HIV-AIDS incidence and prevalence due to preventive measures, like partner reduction, condom use, and improved STD control. Did this monograph have a policy impact, for instance speeding up the implementation of preventive measures? It is not possible to answer this question definitively, as there are many other influences on government actions.

In 1988, the Malawi government prepared its first medium term plan for AIDS control and various donor agencies and researchers became involved in AIDS prevention activities [16]. However, some observers felt that HIV/AIDS prevention activities did not begin in earnest until 1994, following the establishment of a new political regime after the departure of President-for-Life Hastings Banda, an authoritarian ruler who discouraged public discussion of sexuality and HIV [17]. By 1995, AIDS prevention activities had become widespread, and included Anti-AIDS clubs in elementary schools [18], something that would have been unimaginable under President Banda. Modelers often preface a presentation of their worst-case projections with the phrase, “if nothing is done…” Perhaps the projections in Malawi turned out to be accurate because relatively little was done in the early years of the epidemic.

As seen from this study's comparison of the adjusted 1992 HIV prevalence projections with the 2003 NAC official estimates (Figure 2), there is a divergence of the projected high scenario from the actual NAC official estimates around 1995. This suggests that government-initiated preventive measures might have had a modest impact in the mid to late 1990's. This is consistent with the aggressive and high level political commitment to fighting AIDS that was provided by Banda's successor, President Bakili Muluzi, beginning in 1994 [15].

Political sensitivity and openness to epidemiological projections can translate research to practice as demonstrated by success stories of countries like Thailand, Uganda, Kenya and Zimbabwe [19]–[25]. Projections can guide policymakers and ministries of health to formulate successful evidence-based preventive strategies like the 100% condom policy for commercial sex workers, persuasive broadcasting of AIDS awareness messages in televisions and radio, safer sex and “zero-grazing” messages, and school based AIDS education classes in Thailand and Uganda [21], [22], [24]. Strong espousal of preventive measures by non-government organizations, and individual based initiatives in reducing risky sexual behavior can also go a long way in containing HIV-AIDS as can be demonstrated by the success of countries like Kenya, Zimbabwe and Thailand [4]. New approaches, like the use of national health management information systems (HMIS) for HIV surveillance and management, are being proposed, which could potentially improve the linkages between surveillance data, epidemiological projections, and public health programs [26]–[29].

The course of the HIV-AIDS epidemic in Malawi and the social and economic devastation caused by it, despite the presence of reasonably good projections, highlights the need for better co-ordination between epidemiologists, modelers and policy makers. Further research may be useful to examine the difficulties of countries to act more rapidly to implement effective prevention measures, despite the existence of information regarding the possible consequences.

## 

### Conclusion

In 1992, a monograph by Liomba et al, used an epidemiologic modeling approach to project the course of the AIDS epidemic in Malawi. The monograph also suggested some preventive measures to avert a possible catastrophe due to the spread of the epidemic. The actual course of the epidemic in Malawi during the projected years was very close to the high incidence scenario of these projections. The course of the HIV-AIDS epidemic in Malawi and the social and economic devastation caused by it, despite the presence of reasonably good projections and prevention recommendations, highlights the need for better co-ordination between epidemiologists, modelers and policy makers. While warnings from epidemiologists and modelers are not always heeded, this retrospective examination of a simple modeling approach shows that the projections were accurate, and should encourage modelers and epidemiologists to continue their efforts.
